# Interventions through Art Therapy and Music Therapy in Autism Spectrum Disorder, ADHD, Language Disorders, and Learning Disabilities in Pediatric-Aged Children: A Systematic Review

**DOI:** 10.3390/children11060706

**Published:** 2024-06-07

**Authors:** Victoria Martínez-Vérez, Paula Gil-Ruíz, Sara Domínguez-Lloria

**Affiliations:** 1 Department of Didactics of Musical Expression, Art and Dance, Faculty of Education, University of Valladolid, 34004 Palencia, Spain; vita.martinez@uva.es; 2 Department of Corporal and Musical Artistic Expression, Faculty of Education Sciences, Universidad CES Don Bosco, Attached to the Complutense University of Madrid, 28040 Madrid, Spain; pgil@cesdonbosco.com; 3 Department of Special Didactics, Faculty of Education and Sport Sciences, University of Vigo, 36005 Pontevedra, Spain

**Keywords:** art therapy, autism, ADHD, systematic review, music therapy, ADHD

## Abstract

Traditional pharmacological treatments, although effective, often carry potential side effects, which positions art therapy and music therapy as promising non-pharmacological alternatives to alleviate symptoms and improve social, cognitive, and emotional skills without the associated risks. Through a review in the SCOPUS and WOS databases following the PRISMA protocol, a total of 80 articles were analyzed through a series of determined categories and subcategories of analysis. The aim of this study is to evaluate and synthesize the existing evidence on the efficacy and applicability of art therapy and music therapy in the treatment of children with autism spectrum disorder (ASD), hyperactivity disorder (HSDD), developmental language disorders, and language learning difficulties, identifying best practices and key areas for future research. Among the main findings is that art therapy and music therapy have a significant impact on symptomatology, behavior, and communication as well as social, cognitive, and emotional skills in the pediatric populations studied. These therapies are highly valued by the participants with a large majority recognizing their adaptability to different educational and clinical contexts. It is concluded that these therapies have a high potential as viable alternatives or complements to traditional pharmacological treatments, justifying their application and further study in broader therapeutic contexts.

## 1. Introduction

The prevalence of disorders such as attention deficit hyperactivity disorder (ADHD), autism spectrum disorder (ASD), developmental language disorders (SLD), and specific learning difficulties (SLD) has increased in recent years. This upward trend is complex and multivariable, and it includes factors such as evolving diagnostic criteria, increased knowledge and awareness of these conditions, and improved screening methodologies and removal of previous barriers in the diagnostic process [1].

Cybulski et al. [2] report an increase in ADHD and ASD diagnoses based on medical records and parental reports in the United Kingdom, suggesting increased awareness and recognition of these disorders. Similarly, Norbury et al. [3] note that following the removal of certain diagnostic access requirements, the prevalence of TDL is estimated at 7.58% with a 1.22:1 male to female ratio. Specific learning disorders (SLDs) are a group of neurobiological disorders manifested by significant and persistent difficulties in the acquisition and use of academic skills, such as reading, writing, and mathematics. These disorders are not the result of sensory, intellectual, emotional deficits, or an inadequate educational environment. It is noteworthy that these types of disorders frequently co-occur with emotional and behavioral problems [4], oppositional behaviors and socialization difficulties [5], and comorbidities [6]. Furthermore, Zablotsky et al. [7] report a notable 9.5% increase in ASD prevalence among U.S. children aged 3–17 years. The influence of environmental, educational, and sociocultural factors has also been significant.

Art therapy and music therapy have emerged as key interventions in the treatment of disorders such as autism spectrum disorder (ASD), attention deficit hyperactivity disorder (ADHD), Tourette disorder (TDL), and ASD. These therapeutic approaches have gained popularity due to the demand for more personalized and individually tailored treatments, which is crucial in managing the varied manifestations of these disorders [8]. Art therapy offers an alternative means of communication and expression that allows individuals to explore their emotions, develop social skills, reduce anxiety, and increase self-esteem [9]. This approach is particularly useful for children and adolescents with ASD, ADHD, TDL, and ASD, where conventional communication channels are often compromised [10,11]. In contrast, music therapy employs the impact of music on the brain to facilitate communication and expression in addition to improving motor, social, and emotional functions. It can assist in improving behavior regulation and concentration in children with ADHD and promotes verbal and nonverbal communication, potentially minimizing delays in language development and improving the quality of interaction in ASD [8,9,10,11,12].

Recent studies have demonstrated the efficacy of art therapy and music therapy in the treatment of the disorders, emphasizing the need to customize treatments to meet the specific needs of this population [13]. Authors such as López-Hernández et al. [14] propose a music therapy intervention protocol to improve attention, memory, and language in children with dyslalia in the context of TDL and report positive results in this type of intervention. In contrast, art therapies have been demonstrated to enhance attention in children with intellectual disabilities, improve language skills in children with specific language disorders, and contribute to the improvement of reading accuracy and speed in children with learning disabilities [15].

Art therapy and music therapy have emerged as key interventions not only in the management of disorders such as ASD and ADHD but also in the treatment of developmental language disorders and auditory spectrum disorders. They offer significant benefits in improving language articulation and communicative skills, as evidenced by the findings of Livengood-Ordóñez [16] and Guanoluisa [17]. Music therapy has been shown to have a positive effect on language development and communication with notable improvements in language processing and memory observed in patients with dyslalia. Furthermore, specific methodological strategies have been identified as effective in improving speech clarity and fluency. Furthermore, [9] presents a case study that demonstrates how music therapy, when implemented in a community setting, can facilitate emotional expression and reinforce social connections, which are crucial for effective communication. In light of these considerations, the rising prevalence of these disorders, coupled with the advancements in art therapy and music therapy, underscores the necessity for a systematic review to update the scientific information gleaned from the most recent research to the greatest extent possible, given the considerable heterogeneity in the results and research methods employed. A systematic review will enable the synthesis of existing data in a rigorous manner and the assessment of the quality and reliability of studies conducted to date. This will not only provide a solid basis for more informed clinical recommendations but will also identify gaps in the existing literature, directing future research to areas of greatest need and potential. Furthermore, the systematic review will facilitate a more profound comprehension of the potential for these interventions to be optimized and customized for different patient subgroups, thereby maximizing their therapeutic effects in children and adolescents with ASD, ADHD, TDL, and ASD.

## 2. Materials and Methods

For the analysis, we followed the PRISMA protocol that has been registered in the open science framework platform. Following the methodology established by Shaheen et al. [18], our aim is to synthesize existing research and assess its applicability and efficacy in these clinical settings.

We adopted an interpretive approach, allowing a thorough understanding of existing studies, in accordance with the principles described by Page et al. [19]. This approach guided us in the selection and analysis of academic literature, using an observational and retrospective method that is both descriptively and analytically oriented [20].

The literature search was conducted between January and May 2024, using the SCOPUS and Web of Science databases. The most effective search equation used combinations of specific terms applied to the titles, abstracts, and keywords of the documents: (“Art therapy” OR “Music therapy”) AND (“autism spectrum disorder” OR “ASD” OR “ADHD” OR “learning difficulties” OR “language disorders”) AND “children”. This search generated a total of 349 documents in SCOPUS and 406 documents in Web of Science. To reduce the number of documents, a search filter was applied to the period between 2019 and 2023 with the purpose of accessing the most updated information available in both databases, yielding a total of 157 and 133 documents, respectively.

### 2.1. Inclusion and Exclusion Criteria

An inclusion criterion was applied that limited the selection to papers that were research articles only. After this filter, the SCOPUS database submitted 113 papers, while WOS provided a total of 109 papers, resulting in a total of 222 papers. Duplicates were removed, thus reducing the document pool to 176 (*n* = 176), combining the two databases.

The inclusion criteria focused on interventions using art therapy and music therapy in the treatment of disorders such as autism (ASD), attention deficit hyperactivity disorder (ADHD), as well as language development disorders and learning difficulties in the pediatric population, aged 0–14 years.

During the analysis of the documents, those that did not meet the established criteria were excluded. Specifically, 29 articles were eliminated because they did not address the desired age range, 44 papers that did not constitute interventions, including 27 systematic reviews, and 17 studies that presented the design but not the results of the intervention. In addition, 21 papers were excluded because they did not use art therapy or music therapy in the interventions, and two were excluded because they were validations of instruments. As a result, a total of 96 articles were excluded. Finally, a total of 80 articles that met the previously established inclusion criteria were included in the study.

**Figure 1:**
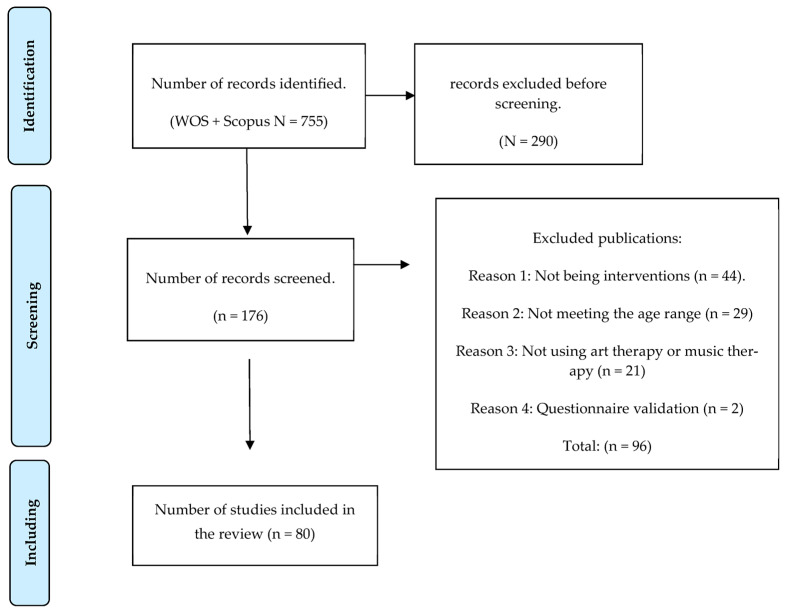
Flow chart according to PRISMA for inclusion and exclusion criteria.

According to the above, the following flow chart was drawn up according to PRISMA standards (Figure 1).

### 2.2. Procedure and Data Analysis

Following an analysis of the identified studies, we proceeded to divide them into four main categories of analysis, which collectively gathered the data from all the selected articles. This is illustrated in Table 1. To mitigate the potential for bias, this analysis process was carried out independently by three researchers.

The analysis of the articles was conducted in accordance with the guidelines established by Para Mayer-Benarous et al. [8] and Geretsegger et al. [21], which indicate that art therapy and music therapy have a significant impact on the pathologies of ASD, ADHD, language development disorders, and learning difficulties. About the age range, the standardized pediatrics age classification was employed, which is determined as follows: newborn (0–6 days), neonate (7–29 days), infant (1–2 years), pre-school (3–5 years), school (6–11 years) and adolescent (12–14 years). Regarding the results of the intervention, the following terms were employed: “high value”, “appropriate value”, and “low value”. The term “high value” was employed when participants perceived that the intervention yielded substantial benefits in relation to the effort invested. The term “appropriate value” was employed when the benefits were deemed to be commensurate with the resources invested. Finally, the term “low value” was employed when participants perceived that the benefits did not justify the resources or time invested.

## 3. Results

### 3.1. Bibliometric Results

As for the geographical location of the studies, Table 2 shows those countries identified in this systematic review.

Upon analysis of the data by continent, it was found that 41 articles were from Asian countries, representing 51.25% of the total. Europe followed with 22 articles, constituting 27.5%, while America contributed 13 articles, which was equivalent to 16.25%. As for the other continents, Africa was represented with three articles, corresponding to 3.75%, and finally Oceania with one article, representing 1.25%.

With respect to productivity, it is observed that the year with the highest number of articles published on this topic was 2020 (*n* = 20), representing a total of 25% of the articles. This was followed by 2022 (*n* = 19), which contributed 23.75% of the articles. The next most productive year was the year 2021 (*n* = 11), contributing 20% of the total production, while the years with the lowest number of articles published on this topic were 2019 (*n* = 14) and 2023 (*n* = 11), representing 17.5% and 13.75% of the total, respectively.

About the journals that host these investigations, a total of 61 different publications can be identified. The Nordic Journal of Music Therapy stands out with six articles (*n* = 6), representing 7.5% of the total. The next most prolific journals are Art Therapy, Arts in Psychotherapy, and The Journal of Music and Human Behavior, which have each contributed four articles (*n* = 4), representing 5% each. Furthermore, the International Journal of Environmental Research and Public Health has three contributions (*n* = 3), representing 3.75%. Finally, and with the least representation, several journals have two articles each (*n* = 2), including Children and Youth Services Review, Frontiers in Psychology, Journal of Music Therapy, Psychiatry Research, and Research in Developmental Disabilities, which collectively represent 2.5% of the total. The remaining journals with only one participation (*n* = 1) represent 61.25%. There is a notable diversity among the authors contributing to academic research. However, a select few have distinguished themselves through their prolific scientific production in specific areas. For instance, Kim Mi-kyung and Lee Esther, both from South Korea, have collaborated on two articles examining the efficacy of art therapy in children with ADHD and ASD. In the Netherlands, Schweizer et al. [22] have published two studies in 2019 that examine the benefits of art therapy on flexibility and social behavior in children with ASD. In the United States, LaGasse et al. [23] made significant contributions to the field of music therapy in the same year, exploring the potential for cognitive improvements in children with ASD. In Italy, Saladino et al. [24,25] have conducted two studies in 2020 and 2021, respectively, investigating the efficacy of art therapy in the context of ASD.

### 3.2. Methodological Aspects

About the methodological approach, 40 studies employed quantitative methodology (50%), 23 utilized qualitative methods (28.75%), and 17 employed a mixed approach (21.25%).

**Figure 2:**
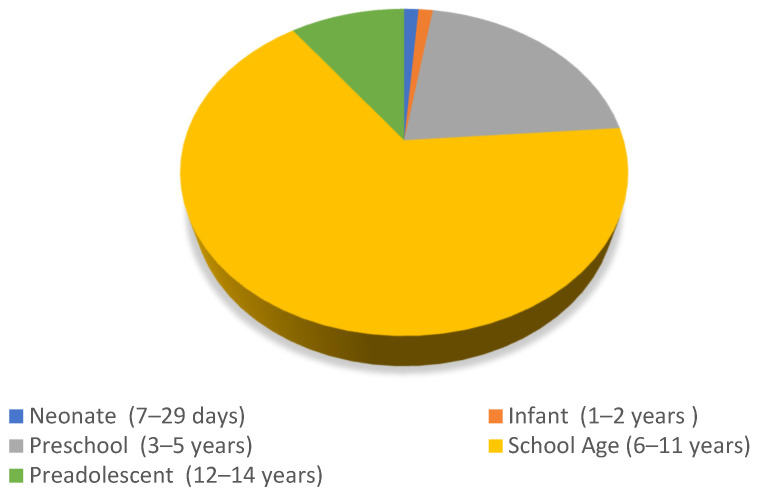
Distribution of participants in the studies according to age range.

Regarding the age range of the participants, 53 studies were conducted with schoolchildren (66.25%), 17 with preschoolers (21.25%), eight with adolescents (10%), and only one study involved infants and neonates (1.25% each; Figure 2).

Regarding the intervention modality, 53 studies employed music therapy (66.25%), while 27 studies utilized art therapy (33.75%).

With respect to the number of participants, two studies with more than 200 participants stand out in the results. One of these studies included 251 participants, while another included 205. Additionally, four studies exceeded 100 participants. Most of the articles analyzed samples of fewer than 100 individuals, representing 91.03% of the total. A noteworthy observation is that thirteen studies with a single participant account for 16.67% of the overall total. In the quantitative approach, questionnaires were employed as the primary instrument in 18 of the 40 interventions, representing 45% of the total. Additionally, systematic recording was employed in 10 studies, representing 25% of the total. Twenty cases involved the use of evaluation instruments and scales, representing 8% of the total number of cases. The medical tests constituted only 10% of the total with only four cases. Regarding qualitative approaches, observation was employed as an instrument in 14 studies (60.86%), interviews in two studies (8.6%), and systematic records in seven cases (30.43%). In mixed studies, questionnaires and observational records were employed as instruments in six studies (35.29%), while assessment tools and scales were used in ten studies (58.82%), and questionnaires and medical tests were used in conjunction with questionnaires in one study (5.8%) as shown in Table 3.

The intervention contexts examined in the studies analyzed include school, clinical, and home settings. Most studies were conducted in clinical settings (*n* = 55), representing 68.75% of the total analyzed. Additionally, school settings play a notable role, with 24 studies included in the analysis, representing 30% of the total. In contrast, home-based interventions are less frequent with only one documented instance representing 1.25% of the total.

### 3.3. Pathologies and Impacts

In the present systematic review, the distribution of pathologies in research in the field of art therapy and music therapy was also analyzed. A total of 80 records were analyzed, and autism spectrum disorder (ASD) was the most prevalent, representing 72.50% of the studies reviewed. The next most prevalent pathology was attention deficit hyperactivity disorder (ADHD), which constituted 22.50% of the cases, followed by language development disorders, which appeared in 2.50% of the cases.

**Figure 3:**
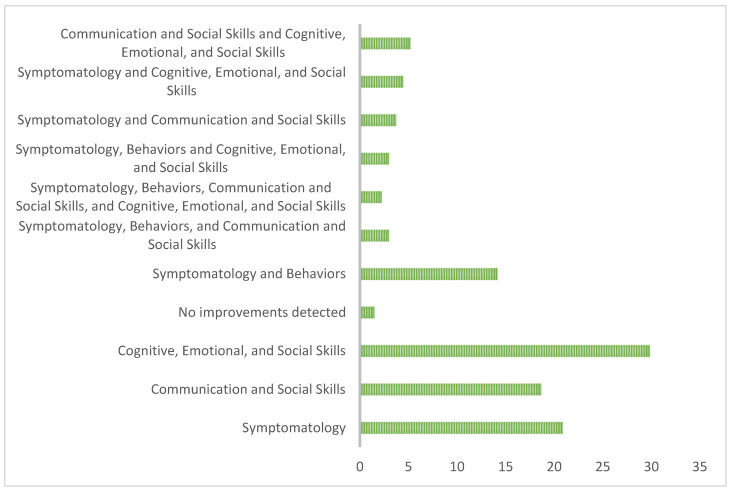
Analysis of the impacts of art therapy and music therapy.

The effects of art therapy and music therapy on the development and well-being of individuals with any of the pathologies are under investigation. The data analysis revealed that improvements in cognitive, emotional, and social skills were the most prevalent with a frequency of 29.85%. The data indicated that 20.90% of the participants reported improvements in symptomatology, while 18.66% reported improvements in communication and social skills. Furthermore, specific combinations of improvements demonstrated notable variations. Improvements in symptomatology and behaviors reached 14.18%, while those combining all the aforementioned areas reached 2.24%. Improvements in symptomatology, behaviors, and communication and social skills registered 2.99%, and improvements in symptomatology and communication and social skills reached 3.73%. Other combinations included improvements in symptomatology and communication and social skills (3.73%), improvements in symptomatology and cognitive, emotional, and social skills (4.48%), and improvements in communication as well as cognitive, emotional, and social skills (5.22%). Meanwhile, 1.49% of the cases reported that no improvements were detected, all this can be seen in Figure 3.

### 3.4. Results of the Interventions

In the present study, a systematic review of the participants’ perceptions of the art therapy and music therapy interventions revealed that 55.56% of the participants perceived the intervention as offering “High Value”. This indicates that the benefits received were deemed to be amply justified by the effort invested. In contrast, 37.78% of the participants evaluated the intervention as “Adequate Value”, indicating that the benefits were commensurate with the resources utilized. Conversely, 6.67% of the participants perceived that the benefits obtained did not justify the resources and time invested, categorizing the intervention as “Low Value”.

## 4. Discussion

The objective of this study is to evaluate and synthesize the existing evidence on the efficacy and applicability of art therapy and music therapy in the treatment of children with autism spectrum disorder (ASD), hyperactivity disorder (HSDD), developmental language disorders, and language learning difficulties. This will involve the identification of best practices and key areas for future research. The variety of countries represented in the systematic review indicates a global interest in the areas of art therapy and music therapy applied to childhood disorders. The prevalence of research in this field in Asia suggests a potential concentration of specialized studies in these countries. In the case of South Korea, the combination of strong government support, a proactive approach to innovation in child welfare and education, and a culture that deeply values the arts and music has contributed to this country’s prominence in the research and application of art therapy and music therapy to treat disorders in children.

The prevalence and awareness of disorders such as autism spectrum disorder (ASD) and attention deficit hyperactivity disorder (ADHD) have increased significantly in recent years. This may have led to an increase in research focused on innovative and non-traditional therapies such as art therapy and music therapy, which are considered promising alternatives or complementary to conventional treatments. A bibliometric study on music therapy revealed a significant increase in the number of publications in recent years, indicating a growing interest in this field. This increase may be related to the increased acceptance and recognition of music therapy as an effective intervention for various conditions, including pediatric conditions. The year 2020, which represents the peak in the number of publications, coincides with the onset of the global COVID-19 pandemic. The pandemic may have significantly affected the care and treatment of children with special needs, driving renewed interest in finding treatment modalities that could accommodate constraints such as confinement and social distancing or offer less invasive and more flexible therapeutic approaches. Furthermore, the years 2020–2022 were distinguished by an expansion in the availability of specific funding to investigate the impact of the pandemic on the mental health and education of children, including those with conditions such as ASD and ADHD. This may be reflected in the increase in studies seeking effective and applicable alternatives in contexts where traditional methods may have been disruptive.

A plethora of journals are devoted to the publication of research on these topics, which indicates the existence of an interdisciplinary field with a growing interest. The notable presence of specific journals such as the *Nordic Journal of Music Therapy* at the forefront of this type of studies suggests the existence of an active and specialized academic community in this field. Conversely, the recurrence of contributions by specific authors in certain countries indicates the existence of centers of excellence or research groups at the vanguard of this field of study.

With respect to methodological aspects, the prevalence of quantitative studies over qualitative and mixed studies suggests a preference for methods that allow statistical generalizations and measurable results. Nevertheless, thanks to the substantial representation of qualitative studies [24,25,48,55,56,57,58,59,60] and mixed methods [61], the literature indicates that a deep understanding of individual experiences and specific contexts is also valued for its ability to deepen understanding of individual experiences, which is crucial for tailoring interventions to the specific needs of individual children [62]. This is of paramount importance to address the inherent complexities of therapeutic interventions involving artistic and musical expressions, which are often subjective and multifaceted [36,63].

Regarding the instruments utilized in qualitative studies to assess the impact of a given intervention, the following instruments stand out as particularly noteworthy: systematic observation through the verbalization rating scale [56], the PLP LENA scales [48], or the FEATS of art therapy [25], as well as in-depth interviews [55].

In quantitative studies, a variety of assessment instruments have been employed to systematically analyze the efficacy and applicability of art therapy and music therapy in the treatment of children with autism spectrum disorder (ASD), attention deficit hyperactivity disorder (ADHD), developmental language disorders, and learning disabilities. To study the impacts on autism spectrum disorder, the following instruments have been preferentially employed: the Childhood Autism Rating Scale (CARS), which is used to assess the severity of autism in children [43]; the Social Communication Questionnaire (SCQ), which is used to measure social communication in children with ASD [26]; and the Autism Spectrum Inventory (ASI), which is used in the assessment of autistic symptoms in several studies [28].

With regard to attention deficit hyperactivity disorder (ADHD), the instruments most frequently employed by researchers are the Behavioral Assessment System for Children (BASC), which allows for the assessment of behavior in children with ADHD [64]; the ADHD Diagnostic Questionnaire (ADHD-RS), which is used to measure the severity of ADHD symptoms [51]; and the Conners Scale, which assesses ADHD through parent and teacher reports [12,13,14,15,16,17,18,19,20,21,22,24,25,26,28,36,43,48,51,55,56,57,58,59,60,61,62,63,64,65,66].

The results indicate a significant concentration among schoolchildren, which may reflect the importance of intervention during the school-age period, which is a crucial stage for social and cognitive development. This underscores the potential value of utilizing this approach as a therapeutic tool during primary education, which is a time when children are developing essential social skills [67].

The efficacy of music therapy in enhancing social responsiveness and communication skills in children with autism spectrum disorder (ASD), attention deficit hyperactivity disorder (ADHD), and language disorders has been demonstrated in multiple studies [39,44,47,68]. Additionally, music therapy has been shown to alleviate the symptoms of these disorders in children [26,43,53,69,70,71], particularly in behaviors [39,44,47,53,66,67,68,69,70,71,72] and cognitive skills [23,29,73,74,75,76].

Art therapy has also been demonstrated to be efficacious in a variety of contexts. For instance, improvements have been observed in symptomatology and behavior [51], communication, and cognitive and emotional skills [26,30,37,58,64,77,78].

Upon examination of the aggregate results, it becomes evident that most of the observed improvements in the interventions pertained to cognitive, emotional, and social skills. These findings indicate that both music therapy and art therapy are effective in multiple dimensions of child development. This underscores the utility of these therapies not only as treatments for specific symptoms but also as holistic approaches to overall child development. The combination of improvements indicates that interventions can have a multifaceted impact, addressing multiple symptoms and developmental domains simultaneously [13,28,79].

Of the articles reviewed, only two indicated that they did not detect improvements or worsening in any symptom or area of ASD. These two articles were both focused on music therapy and art therapy applied to children with ASD. The first of these articles was published in 2021 by Guénoun et al. [79], and the second was published in 2022 by Harvánek et al. [80].

Regarding the modalities of intervention, it can be stated that music therapy is more prevalent than art therapy. Of the 80 studies analyzed, 54 were classified as belonging to the former category. This can be attributed to the perceived effectiveness of music therapy, which has been demonstrated in numerous studies conducted in a variety of clinical contexts. A total of 39 studies employed music therapy, while 16 studies employed art therapy. This perception of greater effectiveness is also due to the participation of a larger number of subjects in the studies, as demonstrated by the research of El-Tellawy et al. [71], which involved 146 children with ASD. Moreover, the accessibility of music, which can be utilized with simple tools such as percussion or voice, renders it more readily available than art therapy, which often necessitates specific and sometimes more expensive resources [62].

About the context of intervention, it is notable that most studies (68.75% or 55) were conducted in clinical settings. This may reflect the need for a controlled environment to effectively evaluate the therapeutic intervention [72]. Nevertheless, studies also indicate the potential for further research in diverse settings, including the school setting, which accounts for 30% (24) of the total. The school setting provides a convenient avenue for accessing therapeutic interventions during school hours, thereby minimizing the disruption to family routines [81]. Finally, a single intervention (1.25%) conducted in the home of a child with ASD who was not in school was identified [13]. Outside the clinical context, interventions can be implemented that are closely aligned with the child’s educational needs. This integration of academic and emotional support in the same setting allows for the implementation of therapies that are tailored to the child’s specific needs [45].

Regarding the diversity of sample sizes, it can be observed that most studies analyze samples of fewer than 100 participants. Conversely, there are instances where the sample size is significantly larger, which could indicate a greater degree of generalizability [53,81,82]. An adequate sample size is crucial to ensure the statistical power needed to detect significant differences, thus avoiding erroneous or insufficient conclusions. A total of 13 single-participant studies (16.25%) were identified, including those by Akinloye et al. [13], Charoenphol et al. [82], Durrani [56], and Harvánek et al. [80]. The following studies were conducted in 2022: Joo [76], Kim and Ho [83], Laubová et al. [55], Lee and Hyang [49], Nielsen and Holck [84], Park [38], Hong [85], and Zaro et al. [42]. The 2020 article highlights the usefulness of case studies for the in-depth exploration of specific therapeutic effects. Despite the limitations of generalization, case studies and small samples are valuable for the exploration of specific phenomena in depth.

A review of the central themes of the studies revealed that autism spectrum disorder (ASD) was the most prevalent, indicating a strong concentration of research on this disorder. In fact, ASD accounted for 73.75% of the studies reviewed. This may reflect the complexity and variety of ASD symptoms, which make creative therapies such as art therapy and music therapy particularly relevant and potentially effective. These therapeutic methods offer an expressive and nonverbal medium that may be more accessible to children with ASD, facilitating improvements in communication and social skills.

Regarding the modality of intervention and the central theme of the papers, a curious aspect is observed. Of the 26 art therapy studies, 50% (13) focus on ADHD, while the remaining 50% (13) focus on children with ASD. The studies on ADHD include [26,28,36,43,61,62,63,64] Chen and Li [30], Cho et al. [64], Kim and Rhee [58], Kim and Ho [83], and Rajabpour Azizi et al. [48]. The studies on children with ASD include Abdulá et al. [26]. The following references were consulted in the preparation of this study: Durrani [56], Fernández-Herrero [77], Guénoun et al. [79], Koo and Thomas [37], Moo and Ho [40], Park [38], Saladino et al. [25], Schweizer et al. [22,32], Thayer and Bloomfield [86], and Wypyszyńska [87].

In contrast, of the 54 music therapy studies, 85% (46) focus on children with ASD [13,23,26,27,29,33,34,35,39,40,41,42,43,44,46,47,48,52,53,54,58,60,66,68,69,70,71,72,73,74,75,76,79,83,84,85,88,89,90,91,92,93,94], 11.3% (6) focus on children with ADHD [22,41,42,52,72,82,94], and 3.7% (2) focus on children with language disorders [14,15,16,17,18,19,20,21,22,23,24,25,26,27,28,29,30,31,32,33,34,35,36,37,38,39,40,41,42,43,44,45,46,47,48,49,50,51,52,53,54,55,56,57,58,59,60,61,62,63,64,65,66,67,68,69,70,71,72,73,74,75,76,77,78,79,80,81,82,83,84,85,86,87,88,89,90,91,92,93,94,95].

These prevalences suggest a possible perceived efficacy bias: art therapy might be seen as more effective for ADHD, whereas music therapy might be considered more effective for children with ASD and language disorders.

In the studies, more than half of the participants (80%, or 64 individuals) perceived the interventions to be of high value, suggesting that the perceived benefits were significant compared to the effort and resources invested. Of these, 79.6% (43) were dedicated to children with ASD, while 18.51% (10) were dedicated to children with ADHD. This finding provides compelling evidence of the perceived effectiveness of the interventions. The high rating of the interventions by the participants serves to reinforce the relevance of continuing and expanding the use of music therapy and art therapy. The perceived benefits underscore the significance of considering participants’ experiences as a pivotal indicator of the efficacy of the interventions (45). Moreover, most participants (80%) indicated that the interventions were adaptable to different contexts, which suggests potential for the implementation of these therapies in diverse educational, clinical, or home settings [64]. Nevertheless, the remaining 20% of participants identified limitations in adaptability, underscoring the necessity for adjustments and customization in the application of these therapies to optimize their efficacy and applicability.

## 5. Conclusions

The results demonstrate the efficacy and favorable perception of art therapy and music therapy in the treatment of children with autism spectrum disorder (ASD), attention-deficit hyperactivity disorder (ADHD), language development disorders, and learning difficulties. The review revealed that autism spectrum disorder (ASD) was the most prevalent, indicating a strong concentration of research on this disorder. The evidence of improvements in multiple areas of development indicates that these therapies offer significant benefits. However, the variability in the perceived value and adaptability of interventions indicates that it is crucial to consider individual and contextual factors when implementing these therapies to maximize their effectiveness and accessibility. The prevalence of clinical settings in therapeutic intervention suggests a preference for controlled settings for the effective evaluation of interventions. The concentration of studies in school-aged children reflects the relevance of these therapies during a vital period for interpersonal and cognitive development. This justifies the integration of music therapy and art therapy in educational and therapeutic programs for children with ASD and related disorders. Nevertheless, we conclude that there are significant advantages to expanding these interventions to settings such as the home and school, which also present unique challenges and opportunities for research and therapeutic practice. Despite the advantages, diversifying intervention settings beyond clinics presents challenges. One such challenge is the need to adapt interventions to less controlled settings, ensuring treatment fidelity in home and school settings.

This review provides a comprehensive overview of current art therapy and music therapy research for children with disorders such as ASD, ADHD, developmental language disorders, and learning disabilities. It indicates both current trends and potential areas for future research.

### Limitations of the Study and Lines of Action

It should be noted that this study has several limitations that require comment. Perhaps the most significant of these is the possibility of expanding the number of studies analyzed. Nevertheless, upon completion of this review, it becomes evident that there has been a notable increase in interest in this subject, as evidenced by the growing number of articles published in scientific journals in recent years. This suggests that music therapy and art therapy, and their relationship with this type of disorder, are becoming increasingly incorporated into the field of music education research. The limited number of studies on ADHD and developmental language disorders indicates a need for further investigation into the potential for adapting or enhancing music therapy and art therapy for these disorders. Research in these areas could benefit a broader and more diverse population, potentially uncovering new therapeutic applications for these techniques.

One of the limitations to consider is that this systematic review is only descriptive, and therefore, we do not obtain a sufficiently critical assessment. It would be advisable to carry out a meta-analysis in the future that would allow us to obtain precise data and carry out a less descriptive analysis.

It is of great interest to systematize these therapies in different contexts and to develop training curricula for therapists that incorporate music and art as tools for the improvement of these types of disorders in the pediatric age group.

## Figures and Tables

**Table 1:** Categories and subcategories of analysis.

Categories	Subcategories
Bibliometric indicators	Geographic location.
	Productivity.
	Publication.
	Authors.
Methodological aspects	Method.
	Age range.
	Intervention modality.
	Participants.
	Instrument.
	Context.
Pathologies and impacts	Type of pathology.
	Improvements detected.
Results of interventions	Participants’ perception.

**Table 2:** Geographical location of the analyzed articles.

Number of Items	Countries	Percentage
11	South Korea and the United States	13.75%
8	China	10%
5	India and Israel	6.25%
4	Iran	5%
3	Italy, Netherlands and United Kingdom	5.08%
2	Spain, France, Cyprus and the Czech Republic	3.75%
1	Kurdistan, Nigeria, Saudi Arabia, Lebanon, Germany, Thailand, Romania, Canada, Egypt, Finland, Taiwan, Ecuador, Norway, South Africa, Denmark, Australia, Poland, Turkey	0.8%

**Table 3:** Quantitative instruments used in the analyzed interventions.

Instruments	Authors
Social Responsiveness Scale-2 (SRS-2)	Abdulá et al. [26]; Mössler et al. [27]; Amel et al. [28]; Yum et al. [29]
Conners children’s behavior questionnaire	Chen et al. [30]; Zhu [31]
OAT (Art Therapy Observation) and EAT-A (Therapist Evaluation)	Schweizer et al. [32]; Schweizer et al. [22]
OAT-A (Child Observation)	Schweizer et al. [22]
Electroencephalography (EEG)	Ramirez-Melendez et al. [33]; Lagasse et al. [23]; Kostilainen et al. [34]; Yum et al. [29]
Combined Raven’s test	Zhu [31]; Rabeyron et al. [35]
Childhood Autism Rating Scale (CARS)	Yum et al. [29]; Al-Ayadhi et al. [36]; Koo and Thomas [37]
5-HT	Park et al. [38]
ADOS and Assessment of the Quality of Relationship (AQR)	Mössler et al. [27]
AMMT (auditory-motor mapping training)	Chenausky et al. [39]
Laban Movement Analysis (LMA)	Moo and Ho [40]
Autism Behavior Checklist (ABC)	Yurteri and Akdemir [41]
Autism Diagnostic Observation Schedule (ADOS-2) and Bayley Scales of Infant and Toddler Development (BSID)	Zaro et al. [42]
BRIEF, CSBQ, and Repetitive Behavior Scale-Revised (RBS-R)	Schweizer et al. [22]
Social Communication Questionnaire (SCQ) and the Strengths and Difficulties Questionnaire (SDQ)	Aithal et al. [43]
DUACS (Voices Together)	Schmid et al. [44]
Conners Scale and the Wisconsin Card Sorting Test (WCST)	Liu et al. [45]
Vineland Social Maturity Scale (VSMS)	Minaabad and Lomar [46]
Parent-Child Movement Scale (PCMS)	Moo and Ho [40]
Functional Near-Infrared Spectroscopy (fNIRS)	Shi et al. [47]
FEATS (Formal Elements Art Therapy Scale) y PPAT (Person Picking an Apple from a Tree Test)	Rajabpour et al. [48]
Impresión Clínica Global (CGI)	Rabeyron et al. [35]
Korean ADHD Rating Scale (K-ADHDDS) and House-Tree-Person Drawing Test (HTP)	Lee and Hyang [49]
Heart Rate Variability (HRV) measurements	Madjar et al. [50]
Pediatric Quality of Life Inventory (PedsQL)	Yurteri and Akdemir [41]
Repetitive Behavior Scale-Revised (RBS-R) and Physical Activity Enjoyment Scale (PACES)	Lakes et al. [51]
Sing and Speak 4 Kids (SS4Kids)	Lim et al. [52]
Electrodermal Activity (EDA)	Akinloye et al. [13]
Social Skills Improvement System (SSIS)	Lee [53]
Vineland Social Maturity Scale (VSMS) and the Test of Language Development (TOLD)	Minaabad and Lomar [46]
Test of Everyday Attention for Children (TEA-Ch)	Lagasse et al. [23]
VISK (Vocabulary Intensive Support Kit) and SBQ (Suicidal Behaviors Questionnaire)	Pater et al. [52]
Wechsler intelligence scale for children (WISC) and Wisconsin card sorting test (WCST)	Zhu [31]
App MAP (versión beta)	Williams et al. [54]
DLP LENA	Laubová et al. [55]

## References

[1] Durkin M. (2019). Increasing prevalence of developmental disabilities among children in the US: A sign of progress?. Pediatrics.

[2] Cybulski L., Ashcroft D., Carr M., Garg S., Chew-Graham C., Kapur N., Webb R. (2021). Temporal trends in annual incidence rates for psychiatric disorders and self-harm among children and adolescents in the UK, 2003–2018. BMC Psychiatry.

[3] Norbury C.F., Gooch D., Wray C., Baird G., Charman T., Simonoff E., Vamvakas G., Pickles A. (2016). The impact of nonverbal ability on prevalence and clinical presentation of language disorder: Evidence from a population study. J. Child Psychol. Psychiatry.

[4] Sofologi M., Kougioumtzis G., Efstratopoulou M., Skoura E., Sagia S., Karvela S., Salli P., Makri E., Bonti E. (2022). Specific Learning Disabilities and Psychosocial Difficulties in Children. Advising Preservice Teachers through Narratives from Students with Disabilities.

[5] Ghislanzoni L., Tobia V., Gambarini A., Rossi E., Tombini G., Ogliari A. (2020). The psychopathological profile of children with specific learning disorders: The point of view of children and their mothers. Eur. J. Spec. Needs Educ..

[6] Mohagheghi M., Pourmohamadreza-Tajrishi M., Shahshahanipour S., Movallali G., Vahedi M. (2022). The Effectiveness of Assertiveness Training on Anxiety Symptoms in School-Age Children with Specific Learning Disorder. J. Rehabil..

[7] Zablotsky B., Black L.I., Maenner M.J., Schieve L.A., Danielson M.L., Bitsko R.H., Blumberg S.J., Kogan M.D., Boyle C.A. (2019). Prevalence and trends of developmental disabilities among children in the United States: 2009–2017. Pediatrics.

[8] Mayer-Benarous H., Benarous X., Vonthron F., Cohen D. (2021). Music therapy for children with autistic spectrum disorder and/or other neurodevelopmental disorders: A systematic review. Front. Psychiatry.

[9] Vrisaba N.A., Yudiharso A. (2021). Systematic Review of Art Therapy in Children with Autism Spectrum Disroder (ASD). Insight J. Pemikir. Dan Penelit. Psikol..

[10] Jeong J., Shim P. (2021). Exploring Art Therapy as a Treatment for Children with Autism Spectrum Disorder. J. Stud. Res..

[11] Gordon T.S. (2020). Fairy-tale therapy as a way to combat phobias. Vestn. Univ..

[12] Chen S.C., Yu B.Y.M., Suen L.K.P., Yu J., Ho F.Y.Y., Yang J.J., Yeung W.F. (2019). Massage therapy for the treatment of attention deficit/hyperactivity disorder (ADHD) in children and adolescents: A systematic review and meta-analysis. Complement. Ther. Med..

[13] Akinloye F.O., Obe O., Boyinbode O. (2020). Development of an affective-based e-healthcare system for autistic children. Sci. Afr..

[14] López-Hernández E., Acosta-Rodas P., Cruz-Cárdenas J., Ramos-Galarza C. (2021). Music therapy intervention for memory, attention, and language in children with dyslalia. Rev. Ecuat. Neurol..

[15] Salgado-Vasco A.F., Rodríguez Barreto A.M. (2022). Experiencia de Musicoterapia Comunitaria en Mujeres con un Embarazo en Conflicto en Tiempos de Covid. ECOS-Rev. Científica Musicoter. Discip. Afines.

[16] Livengood de Sanabria M.D.L.Á. (2022). Musicoterapia en infantes: Funciones cognitivas y emociones. Rev. Cuba. Pediatría.

[17] Guanoluisa M.L.R., Baño E.L.T., Barragán M.F.C. (2023). Estrategias para niños con problemas en el desarrollo del lenguaje. Rev. Dilemas Contemp. Educ. Política Y Valores.

[18] Shaheen N., Shaheen A., Ramadan A., Hefnawy M., Ramadan A., Ibrahim I., Hassanein M., Ashour M., Flouty O. (2023). Appraising systematic reviews: A comprehensive guide to ensuring validity and reliability. Front. Res. Metr. Anal..

[19] Page M., McKenzie J., Bossuyt P., Boutron I., Hoffmann T., Mulrow C., Shamseer L., Tetzlaff J., Akl E., Brennan S. (2021). The PRISMA 2020 statement: An updated guideline for reporting systematic reviews. J. Clin. Epidemiol..

[20] Cienfuegos M.A., y Cienfuegos A. (2016). Lo cuantitativo y cualitativo en la investigación. Un apoyo a su enseñanza. RIDE Rev. Iberoam. Investig. Desarro. Educ..

[21] Geretsegger M., Grant C., Maratos A., Sandford S., Claringbold A., McConachie H., Maskey M., Mössler K., Ramchandani P., Hassiotis A. (2017). International multicentre randomised controlled trial of improvisational music therapy for children with autism spectrum disorder: TIME-A study. Health Technol. Assess..

[22] Schweizer C., Knorth E.J., van Yperen T.A., Spreen M. (2020). Evaluation of ‘Images of Self,’ an art therapy program for children diagnosed with autism spectrum disorders (ASD). Child. Youth Serv. Rev..

[23] Lagasse A.B., Manning R.C.B., Crasta J.E., Gavin W.J., Davies P.L. (2019). Assessing the Impact of Music Therapy on Sensory Gating and Attention in Children with Autism: A Pilot and Feasibility Study. J. Music. Ther..

[24] Saladino V., Sabatino A.C., Sola C.M. (2021). Therapeutic filmmaking and autism spectrum disorder. A case study. Ric. Pedagog. Didatt..

[25] Saladino V., Sabatino A.C., Iannaccone C., Pastorino G.M.G., Verrastro V. (2020). Filmmaking and video as therapeutic tools: Case studies on autism spectrum disorder. Arts Psychother..

[26] Abdulah D.M., Abdulla B.M.O., Liamputtong P. (2023). Impact of short and intensive art-based intervention on symptomatology and social interactions among children with autism spectrum disorder. Clin. Exp. Pediatr..

[27] Mössler K., Gold C., Aßmus J., Schumacher K., Calvet C., Reimer S., Iversen G., Schmid W. (2019). The Therapeutic Relationship as Predictor of Change in Music Therapy with Young Children with Autism Spectrum Disorder. J. Autism Dev. Disord..

[28] Amel A.K., Rahnamaei H., Hashemi Z. (2023). Play therapy and storytelling intervention on children’s social skills with attention deficit-hyperactivity disorder. J. Educ. Health Promot..

[29] Yum Y.N., Lau W.K.-W., Poon K., Ho F.C. (2020). Music therapy as social skill intervention for children with comorbid ASD and ID: Study protocol for a randomized controlled trial. BMC Pediatr..

[30] Chen Y., Li Y. (2022). Art therapy based on painting for classroom interfering behaviors in ADHD intervention. Chin. J. Sch. Health.

[31] Zhu C. (2022). Effects of musicotherapy combined with cognitive behavioral intervention on the cognitive ability of children with attention deficit hyperactivity disorder. Psychiatr. Danub..

[32] Schweizer C., Knorth E.J., Van Yperen T.A., Spreen M. (2022). Exploring Change in Children’s and Art Therapists’ Behavior during ‘Images of Self’, an Art Therapy Program for Children Diagnosed with Autism Spectrum Disorders: A Repeated Case Study Design. Children.

[33] Ramirez-Melendez R., Matamoros E., Hernandez D., Mirabel J., Sanchez E., Escude N. (2022). Music-Enhanced Emotion Identification of Facial Emotions in Autistic Spectrum Disorder Children: A Pilot EEG Study. Brain Sci..

[34] Kostilainen K., Partanen E., Mikkola K., Wikström V., Pakarinen S., Fellman V., Huotilainen M. (2021). Repeated Parental Singing During Kangaroo Care Improved Neural Processing of Speech Sound Changes in Preterm Infants at Term Age. Front. Neurosci..

[35] Rabeyron T., Robledo del Canto J.-P., Carasco E., Bisson V., Bodeau N., Vrait F.-X., Berna F., Bonnot O. (2020). A randomized controlled trial of 25 sessions comparing music therapy and music listening for children with autism spectrum disorder. Psychiatry Res..

[36] Al-Ayadhi L., El-Ansary A., Bjørklund G., Chirumbolo S., Mostafa G.A. (2019). Impact of Auditory Integration Therapy (AIT) on the Plasma Levels of Human Glial Cell Line–Derived Neurotrophic Factor (GDNF) in Autism Spectrum Disorder. J. Mol. Neurosci..

[37] Koo J., Thomas E. (2019). Art Therapy for Children with Autism Spectrum Disorder in India. Art Ther..

[38] Park J.E.J. (2022). A Parent-Focused Creative Approach as a Treatment for a High-Functioning Child with Autism Spectrum Disorder (ASD) in Korea: A Case Study. Int. J. Environ. Res. Public Health.

[39] Chenausky K.V., Norton A.C., Tager-Flusberg H., Schlaug G. (2022). Auditory-motor mapping training: Testing an intonation-based spoken language treatment for minimally verbal children with autism spectrum disorder. Ann. N. Y. Acad. Sci..

[40] Moo J.T.N., Ho R.T.H. (2023). Benefits and challenges of tele-dance movement psychotherapy with children with autism and their parents. Digit. Health.

[41] Yurteri N., Akdemir M. (2019). The effect of music therapy on autistic symptoms and quality of life in children with autism spectrum disorder. Anadolu Psikiyatr. Derg..

[42] Zaro C., Jeon H., Harstad E., Conrad C., Solomon D., Augustyn M. (2020). Questioning a Previous Autism Spectrum Disorder Diagnosis: Can You «Lose» the Diagnosis?. J. Dev. Behav. Pediatr..

[43] Aithal S., Karkou V., Makris S., Karaminis T., Powell J. (2021). Una intervención de psicoterapia con movimiento de danza para el bienestar de niños con un trastorno del espectro autista: Un estudio piloto de intervención. Front. Psicol..

[44] Schmid L., DeMoss L., Scarbrough P., Ripple C., White Y., Dawson G. (2020). An Investigation of a Classroom-Based Specialized Music Therapy Model for Children with Autism Spectrum Disorder: Voices Together Using the VOICSS^TM^ Method. Focus Autism Other Dev. Disabil..

[45] Liu Y., Qian Y., Jiang W.-Q., Zhao Z.-M., Li Y., Chen L., Du Y.-S., Dai Y.-N. (2020). Study on the effect of group music therapy on children with attention deficit hyperactivity disorder. Zhongguo Ertong Baojian Zazhi.

[46] Minaabad M.S., Lomar S.D. (2020). The Effects of Children’s Pedagogical Songs on Social, Linguistic, and Written Skills Development in Children with Autism Spectrum Disorders. J. Client-Centered Nurs. Care.

[47] Shi S., Wang J., Wang Y., Wang H., Zhang Q., Qie S. (2023). Effects of different types of visual music on the prefrontal hemodynamics of children with autism spectrum disorder based on functional near-infrared spectroscopy. Transl. Pediatr..

[48] Rajabpour Azizi M., Rajabpour Azizi Z., Akhavan Tafti M., Mohamadzadeh S. (2022). Comparing the Graphic Performance of Students with and Without SLDs and ADHD Based on FEATS. Art Ther..

[49] Lee E.-H., Hyang L.J. (2022). A Case Study of Cognitive-Behavioral Art Therapy on the Self-Esteem and Aggression of a Child with ADHD. J. Korean Assoc. Dev. Disabil..

[50] Madjar N., Gazoli R., Manor I., Shoval G. (2020). Contrasting effects of music on reading comprehension in preado-lescents with and without ADHD. Psychiatry Res..

[51] Lakes K.D., Neville R., Vazou S., Schuck S.E.B., Stavropoulos K., Krishnan K., Gonzalez I., Guzman K., Tavakoulnia A., Stehli A. (2019). Beyond Broadway: Analysis of Qualitative Characteristics of and Individual Responses to Creatively Able, a Music and Movement Intervention for Children with Autism. Int. J. Environ. Res. Public Health.

[52] Pater M., Spreen M., van Yperen T. (2021). The developmental progress in social behavior of children with Autism Spectrum Disorder getting music therapy. A multiple case study. Child. Youth Serv. Rev..

[53] Lee J.K. (2021). Music Therapy for Children·Adolescents with Autism Spectrum Disorders and the Effect on Social Skills and Depression. J. Learner-Centered Curric. Instr..

[54] Williams T.I., Loucas T., Sin J., Jeremic M., Aslett G., Knight M., Fincham-Majumdar S., Liu F. (2021). A randomised controlled feasibility trial of music-assisted language telehealth intervention for minimally verbal autistic children—The MAP study protocol. Pilot Feasibility Stud..

[55] Laubová J., Li J., Kučera M., Kantor J. (2023). Případová studie vlivu hudební senzomotorické integrační terapie na řeč a vývoj chlapce s poruchou autistického spektra. Rehabilitacia.

[56] Durrani H. (2020). Art Therapy’s Scope to Address Impaired Attachment in Children with ASD and Comorbid SID. Art Ther..

[57] Kim J.-M., Park E.-S. (2020). The Concept Mapping of Art Therapist’s Perception about the Factors of Building Therapeutic Relationships with Children with ASD. Korean J. Arts Ther..

[58] Kim M., Rhee E. (2020). A Analysis of Mediation Effect of Music-Oriented Convergent Arts Therapy Program for Improving Problem Behavior in ADHD Children. Korean J. Arts Educ..

[59] Thompson G.A., Shanahan E.C., Gordon I. (2019). The role of music-based parent-child play activities in supporting social engagement with children on the autism spectrum: A content analysis of parent interviews. Nord. J. Music. Ther..

[60] Borzabadi Farahani Z., Rahgoi A., Fallahi-Khoshknab M., Hosseinzadeh S. (2023). The Effect of Art Therapy (Mandala Coloring) on the Attention Level of Children with Attention Deficit/Hyperactivity Disorder. Art Ther..

[61] Dunphy K., Mullane S., Jacobsson M. (2014). The effectiveness of expressive arts therapies: A review of the literature. Psychother. Couns. J. Aust..

[62] Bradt J. (2021). Where are the mixed methods research studies?. Nord. J. Music Ther..

[63] Brooks D. (2003). A History of Music Therapy Journal Articles Published in the English Language. J. Music Ther..

[64] Cho H., Jeon H.-J. (2020). Effects of ROCF drawing-integrated cognitive behavioural art therapy on ROCF drawing performance and changes in core symptoms and problem behaviours in children with ADHD. J. Cogn. Dev. Interv..

[65] Kamioka H., Tsutani K., Yamada M., Park H., Okuizumi H., Tsuruoka K., Honda T., Okada S., Park S., Kitayuguchi J. (2014). Effectiveness of music therapy: A summary of systematic reviews based on randomized controlled trials of music interventions. Patient Prefer. Adherence.

[66] Ke X., Song W., Yang M., Li J., Liu W. (2022). Effectiveness of music therapy in children app with autism spectrum disorder: A systematic review and meta-analysis. Front. Psychiatry.

[67] Dănciulescu T., Zaharia A. (2023). Piano with a twist: A pilot study exploring the preliminary effects of a piano therapy program for children with autism spectrum disorder. Arts Psychother..

[68] Bharathi G., Venugopal A., Vellingiri B. (2019). Music therapy as a therapeutic tool in improving the social skills of autistic children. Egypt. J. Neurol. Psychiatry Neurosurg..

[69] Cibrian F.L., Madrigal M., Avelais M., Tentori M. (2020). Supporting coordination of children with ASD using neurological music therapy: A pilot randomized control trial comparing an elastic touch-display with tambourines. Res. Dev. Disabil..

[70] Dvir T., Lotan N., Viderman R., Elefant C. (2020). The body communicates: Movement synchrony during music therapy with children diagnosed with ASD. Arts Psychother..

[71] El-Tellawy M.M., Ahmad A.R., Saad K., Alruwaili T.A.M., AbdelMoneim I.M., Shaaban I., Alinad A.K.M., Albulayhid S.B.H., Khalaf S.M. (2022). Effect of hyperbaric oxygen therapy and Tomatis sound therapy in children with autism spectrum disorder. Prog. Neuro-Psychopharmacol. Biol. Psychiatry.

[72] Lee L., Lin H.-F. (2023). The influence of music technology on the academic behavior of preschool children with autism spectrum disorder. Eurasia J. Math. Sci. Technol. Educ..

[73] Attar N., Al-Hroub A., El Zein F. (2022). Effects of Three Music Therapy Interventions on the Verbal Expressions of Children with Autism Spectrum Disorder: A Combined Single-Subject Design. Front. Psychol..

[74] Blauth L., Oldfield A. (2022). Research into increasing resilience in children with autism through music therapy: Statistical analysis of video data. Nord. J. Music. Ther..

[75] Carpente J.A., Gattino G.S., Berrones Cortez G.X., Kelliher M., Mulholland J. (2022). Convergent Validity for the Individual Music-Centered Assessment Profile for Neurodevelopmental Disorders. J. Music. Ther..

[76] Joo L.E. (2022). An Art therapy Case Study on the Interaction Between Children with Autism Spectrum Disorder and Their Mothers. Korean, J. Art Ther..

[77] Fernandez Herrero J. (2023). Group creative activities to improve social and emotional competencies of youngsters with ASD. Artseduca.

[78] Feng H., Mahoor M.H., Dino F. (2022). A Music-Therapy Robotic Platform for Children with Autism: A Pilot Study. Front. Robot. AI.

[79] Guénoun T., Tiberghien C., Juteau A. (2021). Videodrama: Cartoon-based therapeutic mediation for children with autism spectrum disorders. Neuropsychiatr. L’enfance L’adolescence.

[80] Harvánek R., Kučera M., Du J., Li J., Kantor J. (2022). The effect of musical sensorimotor integrative therapy on the speech of a child with autism. Rehabil. Fyzikalni Lek..

[81] Simpson J., Atkinson C. (2019). The Role of School Psychologists in Therapeutic Interventions: A Systematic Literature Review. Int. J. Sch. Educ. Psychol..

[82] Charoenphol C., Tayrattanachai N., Chiengchana N. (2019). The effects of parent-child interactive music therapy on sentence verbalisation in a child with autism spectrum disorder: A case study. Malays. J. Music..

[83] Kim Y., Ho J.S. (2021). A Disordered Child of Hyperactivity and Attention Deficit for the Study of Art Therapy and Single Example Case: In the Center of a Preschool Child. J. Parent Educ..

[84] Nielsen J.B., Holck U. (2020). Synchronicity in improvisational music therapy–Developing an intersubjective field with a child with autism spectrum disorder. Nord. J. Music. Ther..

[85] Hong Y.-N. (2021). Environment of children’s music therapy from the perspective of ecology. J. Environ. Prot. Ecol..

[86] Thayer F., Bloomfield B.S. (2021). An evaluation of a developmental individual differences relationship-based (DIR^®^)- creative arts therapies program for children with autism. Arts Psychother..

[87] Wypyszyńska J., Zaboklicka N., Stachura M., Sito Z., Męcik-Kronenberg T. (2021). Opinions of Parents of children with autism spectrum disorders on art therapy in the improvement of their functioning. Wiad. Lek..

[88] Liu T., Schultz B.G., Dai D., Liu C., Lense M.D. (2022). Parent-child nonverbal engagement during read versus sung book-sharing in preschoolers with and without ASD. Psychol. Music..

[89] Marom M., Gilboa A., Bodner E. (2020). Countertransference responses of one music therapist to autistic echolalia. Nord. J. Music. Ther..

[90] Nell N., de Villiers F., Griessel D.J. (2022). Reflections on Teaching Piano to Young Children Diagnosed with Autism Spectrum Disorders. Muziki.

[91] Redondo Pedregal C., Heaton P. (2021). Autism, music and Alexithymia: A musical intervention to enhance emotion recognition in adolescents with ASD. Res. Dev. Disabil..

[92] Salomon-Gimmon M., Elefant C. (2019). Development of vocal communication in children with autism spectrum disorder during improvisational music therapy. Nord. J. Music. Ther..

[93] Yoo G.E., Im J.Y., Ha E.J. (2021). Feasibility of synchronous videoconferencing interactive singing program for children with autism spectrum disorder during COVID-19. J. Music. Hum. Behav..

[94] Lim H.A., Ellis E.M., Sonnenschein D. (2022). Effect of Sing and Speak 4 Kids: An Online Music-Based Speech and Language Learning Game for Children in Early Intervention. Child Lang. Teach. Ther..

[95] Forti S., Colombo B., Clark J., Bonfanti A., Molteni S., Crippa A., Antonietti A., Molteni M. (2020). Soundbeam imitation intervention: Training children with autism to imitate meaningless body gestures through music. Adv. Autism.

